# Prior brief meditation reduces distractor inhibition during cognitive interference

**DOI:** 10.3389/fpsyg.2024.1445327

**Published:** 2024-10-01

**Authors:** Masahiro Fujino, Yuuki Ooishi, Yoshiyuki Ueda, Norimichi Kitagawa, Michio Nomura

**Affiliations:** ^1^NTT Communication Science Laboratories, NTT Corporation, Kanagawa, Japan; ^2^Graduate School of Education, Kyoto University, Kyoto, Japan; ^3^Institute for the Future of Human Society, Kyoto University, Kyoto, Japan; ^4^BKC Research Organization of Social Sciences, Ritsumeikan University, Shiga, Japan; ^5^Yoshika Institute of Psychology, Shimane, Japan

**Keywords:** mindfulness meditation, focused attention meditation, open monitoring meditation, distractor inhibition, mere exposure effect, cognitive interference

## Abstract

**Background:**

Mindfulness meditation, comprising focused attention and open monitoring meditations, has been shown to enhance performance on cognitive interference tasks. While this enhancement has been considered not to result from distractor inhibition, no empirical evidence has been provided through behavioral data. In this study, we investigated whether 30-min interventions of focused attention and open monitoring meditations could reduce distractor inhibition in 72 meditation-naïve participants divided into focused attention meditation, open monitoring meditation, and control groups.

**Methods:**

We employed a task set that combined a cognitive interference task with subsequent preference judgment and surprise recognition tasks, utilizing the mere exposure effect paradigm, along with state and trait questionnaires. The mere exposure effect shows that repeated exposure to face images increases one’s preference for them. However, this effect is reduced if participants consciously or unconsciously try to inhibit the face images during stimulus processing. In the cognitive interference task, they judged the direction of the letter superimposed on a distractor face image. In the subsequent preference judgment task, they were asked to rate the preference of face images, half of which were presented in the interference task and the remaining half were not presented. We hypothesized that inhibiting face images presented as distractors would lead to a decrease in preference for them.

**Results and discussions:**

We found that the mere exposure effect was observed in focused attention meditation and open monitoring meditation groups but not in the control group, indicating that compared to the control, focused attention and open monitoring meditations reduce inhibition processes for distractors during cognitive interference tasks. Furthermore, we found a positive correlation between the intensity of the mere exposure effect and state relaxation before the cognitive interference task as well as a negative correlation between the intensity of the mere exposure effect and state anxiety in the focused attention meditation group, but not in the open monitoring meditation group. This suggests that the processes of reducing inhibition in focused attention and open monitoring meditations differ. Our findings contribute to understanding the attentional mechanisms underlying mindfulness meditation during cognitive interference.

## 1 Introduction

Over the past few decades, several studies have indicated that mindfulness meditation improves performance in a cognitive interference task such as an Attention Network Test and a Stroop task (Ainsworth et al., 2013; Gallant, 2016; Moore and Malinowski, 2009), where participants engage in target-related cognitive tasks amidst interference from distractors. In the field of cognitive psychology, improving performance in cognitive interference tasks is thought to be achieved by consciously or unconsciously inhibiting distractors (van Moorselaar and Slagter, 2020). However, it is said that mindfulness involves paying non-judgmental and non-reactive attention to experiences in the present moment rather than inhibiting them (Chambers et al., 2009; Kabat-Zinn, 1990). In fact, some studies of surveys have demonstrated that the mindfulness trait is negatively correlated with the tendency to inhibit emotional expression and thought (Baer et al., 2006; Iani et al., 2019). A brain imaging study has also revealed that mindfulness meditation induces a deactivation of the medial prefrontal cortex, which has inhibitory projections to the amygdala (Quirk and Gehlert, 2003), without deactivating the amygdala in experienced meditators during emotional processing (Taylor et al., 2011). Furthermore, another brain imaging study (Kozasa et al., 2012) found that meditation practitioners performed the Stroop task with the same performance level as non-practitioners but with lower activity in attention-related brain regions, including the right medial frontal gyrus, middle temporal gyrus, precentral and postcentral gyri, and the lentiform nucleus, during incongruent conditions. They suggested that meditation may reduce interference from distractions and enhance brain efficiency in attention and impulse control. Thus, it has been considered that, in cognitive interference tasks, mindfulness meditation would improve performance through attention regulation strategies other than distractor inhibition. However, to the best of our knowledge, no study has provided empirical behavioral evidence for the view that mindfulness meditation reduces inhibition processes during cognitive interference tasks. Behavioral evidence is required to develop a more comprehensive understanding of the neural, biological, and psychological mechanisms of mindfulness meditation and to clarify the mechanism of the attention regulation strategy in cognitive interference.

Mindfulness meditation typically comprises focused attention meditation (FAM) and open monitoring meditation (OMM). FAM entails the voluntary focusing of attention on a chosen object (Lutz et al., 2008). Typical instructions for FAM require people to focus on a chosen object and reorient it whenever they recognize that their attention is being captured by distractors. Importantly, the instructions do not mention the inhibition of distractors. Meanwhile, OMM involves non-reactive moment-to-moment monitoring of the content of one’s experiences (Lutz et al., 2008). Since this monitoring does not create any explicit focus, OMM does not distinguish between selected and deselected objects (Lutz et al., 2008). Hence, there should be no distractors to be inhibited during OMM.

Considering these characteristics of FAM and OMM, the inhibition related to stimulus processing would be affected by FAM and OMM. While several studies have demonstrated that both FAM and OMM interventions improved the performance in cognitive interference task and attention regulation tasks (Ainsworth et al., 2013; Mrazek et al., 2012), none have shown that both reduce inhibition processes during cognitive interference tasks using behavioral experiments. One reason is that although previous studies employed tasks to evaluate cognitive interference performance, it could not determine whether inhibition occurs or not. For instance, if performance on the Flanker task is high, it remains unclear whether this is due to distractor inhibition or simply not being affected by them without inhibition. To address the issue, our study applied the procedure of the mere exposure effect, which involves an increase in preference for an object after repeatedly being exposed to it (Zajonc, 2001). Zajonc (1968) demonstrated that as the number of exposures to face images increases from 0 to 25, the preference for those faces also increases. It is also known that this effect reverses, disappears, or weakens depending on the degree of the mere exposure effect of the distractor and suppression of the distractor (Huang and Hsieh, 2013), when the object repeatedly presented is a distractor of the concurrent task (De Vito et al., 2017; Fenske and Raymond, 2006; Inoue and Sato, 2017; Raymond et al., 2003). Fenske and Raymond (2006), based on their findings (Raymond et al., 2003; Raymond et al., 2005), proposed that when a distractor competes for control over response during cognitive interference, distractor inhibition is applied, resulting in a reduced preference for the distractor. Applying these findings, we investigated whether FAM and OMM interventions induce the mere exposure effect as evidence of inhibition reduction, even when the object is presented as a distractor.

We hypothesized that the FAM intervention would improve performance on the cognitive interference task, which requires ignoring a distractor to respond accurately, compared to the control condition (i.e., relaxed without any intentional attention control). For the attentional regulation strategies during cognitive interference, target facilitation (i.e., the prioritization of goal-relevant information) and distractor inhibition (i.e., the inhibition of goal-irrelevant information), which are part of the top-down attention regulation strategy, were distinct mechanisms based on different neural substrates (Noonan et al., 2016; Xie et al., 2020). If FAM mainly enhances target facilitation, the mere exposure effect on the distractor object would be observed after the FAM intervention, because participants would have a reduced need to inhibit distractors. Meanwhile, if FAM enhances distractor inhibition, the mere exposure effect would not be observed. Furthermore, OMM would also improve the performance of the cognitive interference task compared to the control condition. OMM does not distinguish between selected and deselected objects (Fujino et al., 2018; Lutz et al., 2008). Consequently, there may no longer be anything that needs to be inhibited after the OMM intervention. Therefore, the mere exposure effect on the distractor object would be observed after the OMM intervention.

## 2 Materials and methods

### 2.1 Participants

The study predetermined the sample size as 72 based on the necessity of counterbalancing and our laboratory’s experience. The required minimum sample size was 42 participants using G*Power (version 3.1.9.2; Faul et al., 2007) for an analysis of variance (ANOVA) (repeated measures, within-between interaction), using an effect size of 0.25, a significance level of 0.05, and a power of 0.8. To investigate the mere exposure effect with participants who appropriately completed the cognitive interference tasks, we established an exclusion criteria (mentioned in the data analysis section) and checked for them before analyzing the results of the mere exposure effect. When a participant was excluded, we recruited another one. Seventy-eight undergraduate and graduate students from Kyoto University, who were naïve to meditation practices, were recruited. We excluded one participant due to a procedural error and five participants due to reaction times (See 2.7 Data analysis). Finally, data from 72 participants were analyzed (36 females, 36 males; *M*_age_ = 20.03 years, SD = 2.00).

### 2.2 Intervention

We prepared three conditions, namely the FAM, OMM, and a control, as experimental interventions. There is some debate as to how much meditation training can produce these effects. Previous research revealed that a 10-min intervention of FAM, OMM, and relaxation reduced the state of anxiety following the intervention compared to baseline, in which there was no significant difference across groups (Ainsworth et al., 2015). In comparison, Ooishi et al. (2021) found that FAM increased para-sympathetic nerve activity, while OMM increased sympathetic nerve activity and decreased salivary cortisol levels with a 30-min meditation training developed by Fujino et al. (2019). Accordingly, 30-min interventions would be expected to have different effects on the inhibition processing in cognitive interference tasks. Therefore, for FAM and OMM conditions, we used 30-min voice instructions (Fujino et al., 2019). The instructions consisted of five parts: “overview of instruction,” “how to assume the correct posture,” “how to breathe,” “how to perform the mental exercise,” and “how to finish.” Each part consisted of multiple instances of voice guidance and periods of mental practice, presented alternately. For the mental exercise of FAM, participants learned to pay attention to their breathing and return their attention to it when they noticed that their minds had become distracted, all with their eyes closed. For the mental exercise of OMM, participants learned to allow their breathing to occur naturally and be aware of the sensations when it occurred. They also learned to be aware of distractions without judgment or criticism when they noticed that their minds were distracted, and how to feel the impact of those distractions on their bodies, all with their eyes closed. For the control condition, participants were instructed to relax on a sofa with some landscape or animal photobooks. All the audio instructions were recorded by the same meditation instructor. See more details in the work of Fujino et al. (2019), which include both Japanese and English scripts.

### 2.3 Questionnaire

To examine the effect of interventions, questionnaires on the state of anxiety [state section of the State–Trait Anxiety Inventory, STAI; developed by Spielberger et al. (1970) and translated into Japanese by Shimizu and Imae (1981)] and relaxation [self-report measure to assess relaxation effects, S-MARE; developed by Sakakibara et al. (2014), comprising three subscales, namely physiological tension, psychological relaxation, and anxiety] were performed before and after the interventions (hereafter, they are called 1st and 2nd state questionnaires, respectively). Moreover, we asked participants to complete three questionnaires on their traits to confirm that there are no differences in the latter between groups at the end of the experiment. The first one is the mindfulness trait [Five Facet Mindfulness Questionnaire, FFMQ; developed by Baer et al. (2006) and translated into Japanese by Sugiura et al. (2012), comprising five subscales: observing, non-reactivity, nonjudging, describing, and acting with awareness]. The second one is social phobia [Social Phobia Scale, SPS; developed by Mattick and Clarke (1998) and translated into Japanese by Kanai et al. (2004)], as a previous study demonstrated that socially anxious individuals preferentially allocate their attention toward threatening faces compared to non-anxious controls (Bantin et al., 2016). The third one is optimism [the Life Orientation Test-Revised, LOT-R; developed by Scheier et al. (1994) and translated into Japanese by Sakamoto and Tanaka (2002)], as a previous study showed that participants scoring high on dispositional optimism tended to gaze longer at joyful faces (Peters et al., 2016).

### 2.4 Apparatus

Instructions and visual stimuli were displayed on a monitor (Dell P992) with a resolution of 1,360 × 768 pixels using MATLAB (MathWorks, USA) using the Psychophysics Toolbox Version 3.0.12 (Brainard, 1997; Pelli, 1997). Participants sat on a chair with their heads positioned on a chin rest at a distance of approximately 40 cm from the monitor.

### 2.5 Face images

Images of angry, neutral, and smiling faces of 12 females and 12 males were chosen from the Kokoro Research Center facial expression database (Ueda et al., 2019) and were classified into three sets of three facial expressions of four female and four male face images. Face images in two of the three sets were used in cognitive interference tasks before and after the intervention (hereafter called the 1st and 2nd cognitive interference tasks, respectively) as exposure stimuli. Furthermore, the neutral face images presented in the 2nd cognitive interference task and those of the other set, which were not presented in both the cognitive interference tasks, were used as the test stimuli in the following preference judgment task. Notably, while three types of facial expressions were used in the cognitive interference task, only neutral faces were used in the preference judgment task. This design was based on previous research indicating that preference was significantly higher when participants were exposed to various facial expressions compared to a single facial expression (Kawakami and Yoshida, 2011). The same neutral face images were also used as test stimuli in the subsequent surprise recognition task, which aimed to confirm whether participants recognized the face images during the cognitive interference task. The size of all face images was 19.2° in width and 22.6° in height. The face image sets were counterbalanced across participants.

### 2.6 Procedure

**Figure 1:**
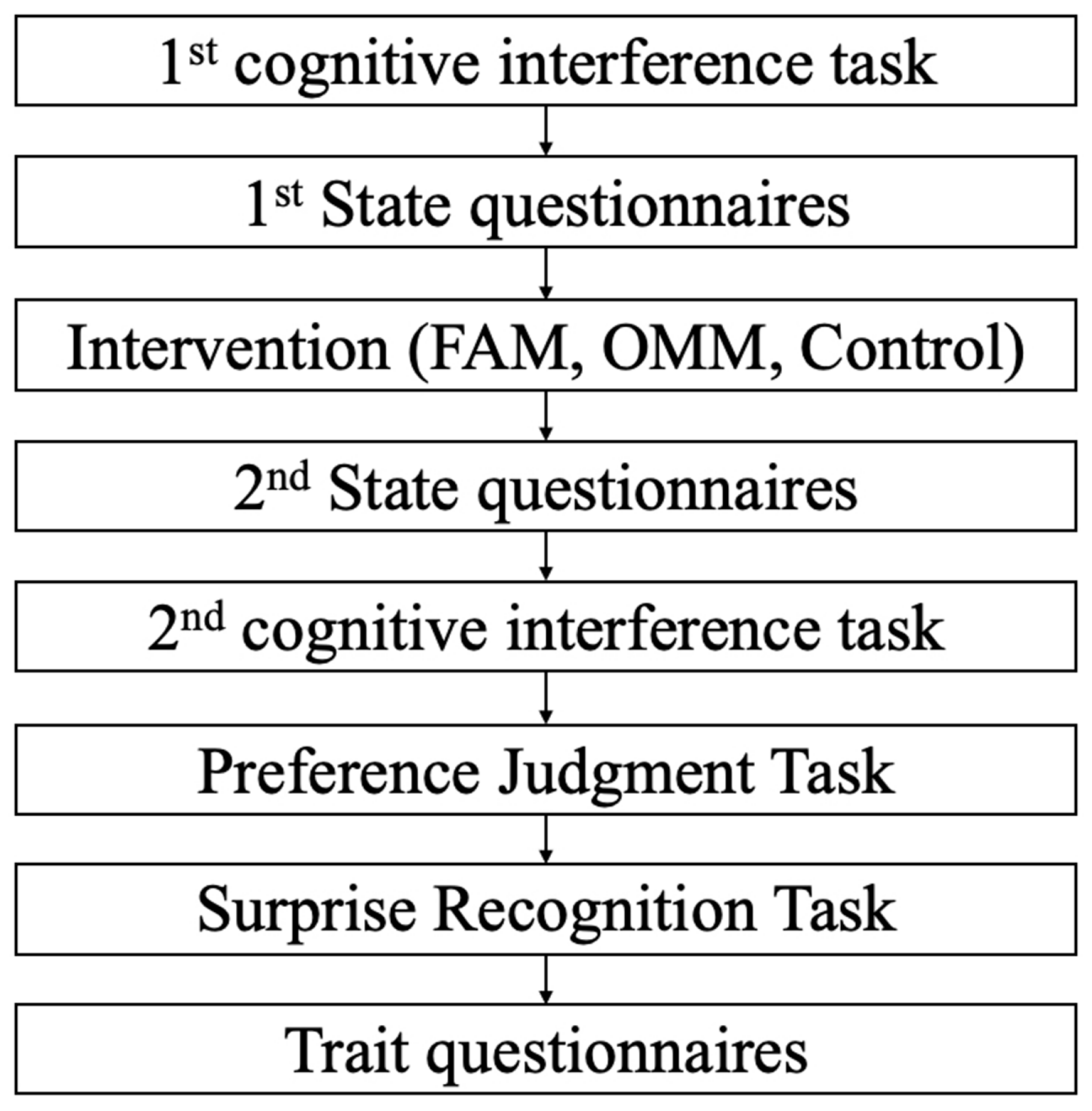
Experimental procedure.

Participants were randomly assigned to three groups, FAM, OMM, and the control group, each of which consisted of 24 participants. Participants in these groups received one corresponding intervention among FAM, OMM, and control conditions. Experimenters, who were unaware of the purpose of this study, did not know which condition the participants were assigned to because the experimenters only pressed the button to start the prepared audio instruction. Participants did not know what kind of conditions they were involved in either (i.e., double-blind). Participants performed the cognitive interference task before and after the intervention, followed by the preference judgment and surprise recognition tasks (Figure 1). They answered two state questionnaires just before and after the intervention and three trait questionnaires at the end of the experiment.

**Figure 2:**
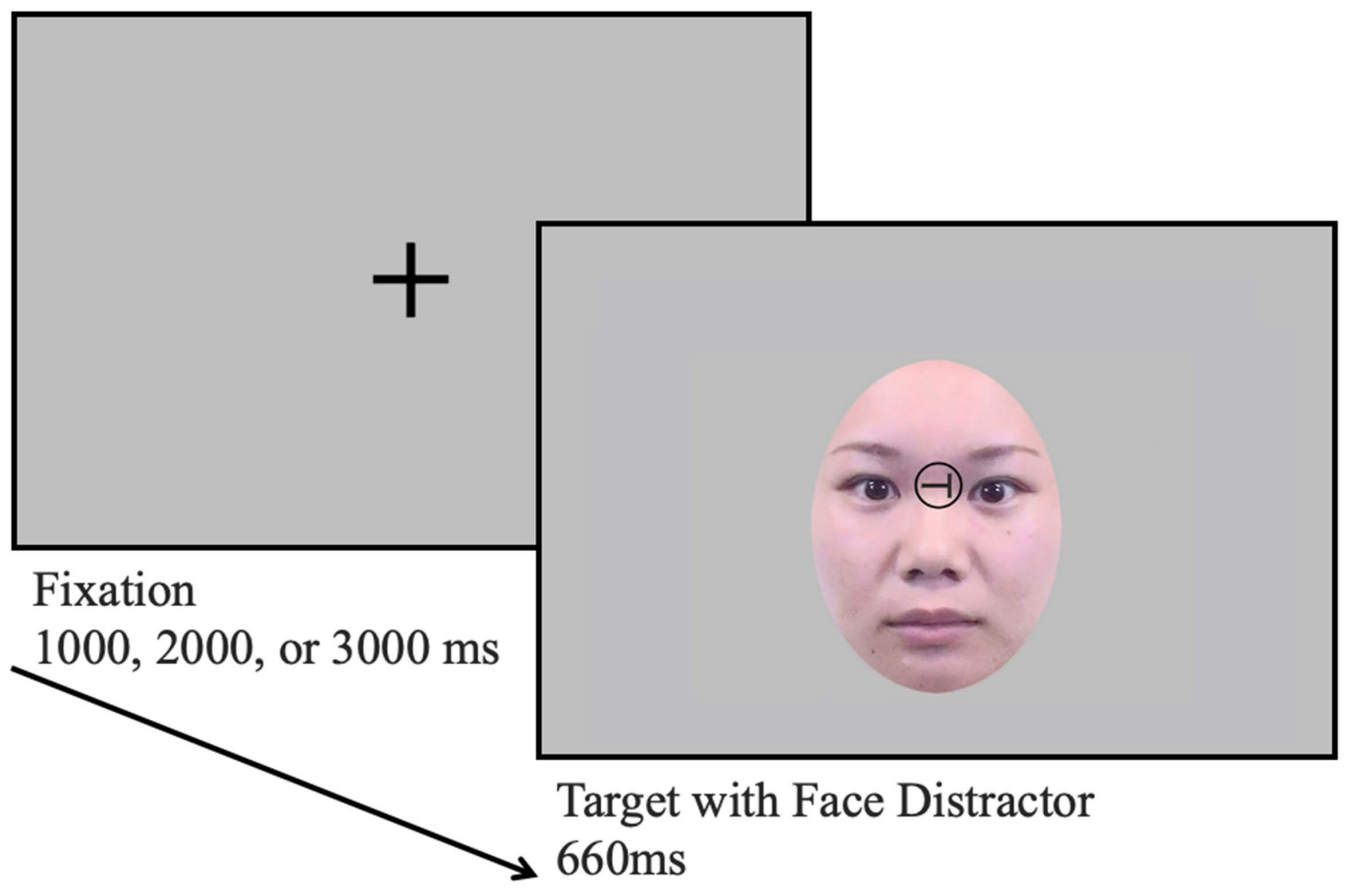
The Flow of the cognitive interference task. The character used as targets were either “T” or “Y,” and they could be oriented upwards, downwards, to the right, or to the left. Participants pressed the button to indicate the orientation of the character. The image was reproduced from an existing publication in Ueda et al. (2019).

For the cognitive interference task, we developed a new task in the preliminary experiment of this study (see more details about the preliminary experiment in the Supplementary material). The cognitive interference task should comprise a target that the participants have to respond to and a distractor to ignore, examining individuals’ preference for distractors in the preference judgment task. By making distractions irrelevant to the object, the extent to which participants inhibit the distraction could be examined. Moreover, since distractors should be salient to ensure the mere exposure effect, a face image was used as a distractor. A letter (“T” or “Y”) placed between the eyes of the face was used as a target (Figure 2). In the beginning of the trial, the fixation appeared in the center of the display for 1,000, 2000, or 3,000 ms randomly; subsequently, the letter and face image appeared. Participants were asked to discriminate the orientation of the letter (up, down, left, or right) and answer it using a keyboard as soon and accurately as possible while ignoring the facial expressions. Both the letter and face image were presented for 660 ms, irrespective of the participants’ key press. There were six practice trials followed by seven experimental blocks, each comprising 24 trials. In each block, the three facial expressions of four females and four males were presented once in random order. Therefore, participants were exposed to each person’s face 21 times (i.e., three facial expressions × seven blocks).

**Figure 3:**
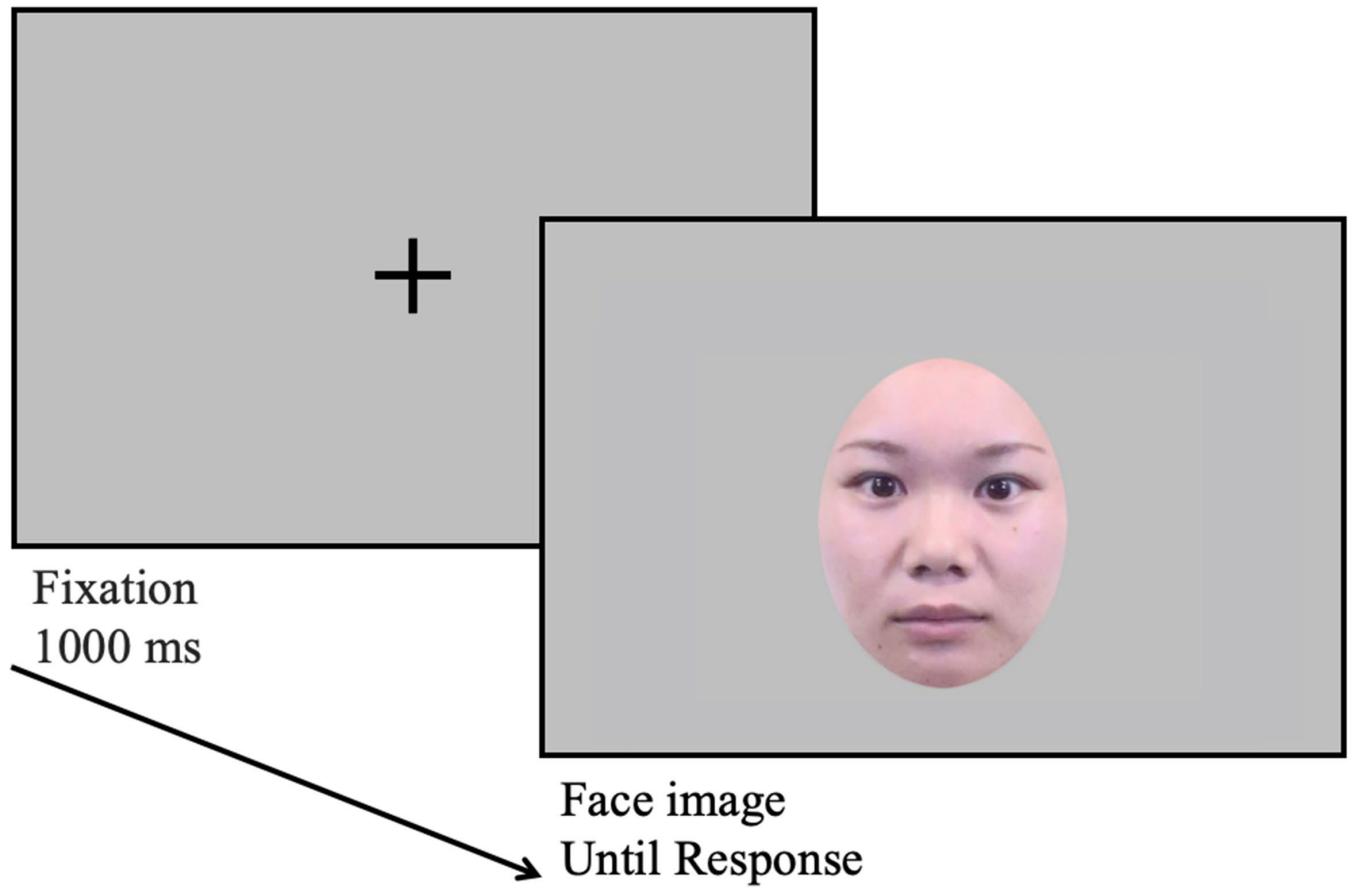
The flow of both preference judgment and surprise recognition tasks. In the preference judgment task, participants pressed the button to indicate the preference of the face image on a nine-point Likert scale (1 = very unattractive, 5 = neutral, and 9 = very attractive). In the surprise recognition task, they pressed the button to indicate whether the face image was presented in the 2nd cognitive interference task. The image was reproduced from an existing publication in Ueda et al. (2019).

In the preference judgment and surprise recognition tasks (Figure 3), after the fixation appeared in the center of the display for 1,000 ms, a neutral face appeared. In the preference judgment task, participants were asked to rate their preference on a nine-point Likert scale (1 = very unattractive, 5 = neutral, and 9 = very attractive). In the following surprise recognition task, they judged whether it was presented in the 2nd cognitive interference task. The face image was presented until the participant responded. There were 16 trials conducted; half were presented in the 2nd cognitive interference task and the other half were novel to the participants. Participants were given no information about the preference judgment task until they finished the 2nd cognitive interference task and about the surprise recognition task until they finished the preference judgment task.

### 2.7 Data analysis

For the analysis of trait questionnaires, mean scores were calculated in each condition (three interventions: FAM vs. OMM vs. control), and an analysis of variance (ANOVA) with one between-subject factor (intervention) was conducted to examine whether there were any differences in personality traits. For the analysis of state questionnaires, mean scores were calculated in each condition (3 interventions: FAM vs. OMM vs. control × 2 timings: 1st vs. 2nd), and a 3 × 2 repeated-measures analysis of variance (rm-ANOVA) with one between-subject factor (intervention) and one within-subject factor (timing) was conducted to examine the effect of interventions.

For the behavioral data, the correct ratio and reaction time in the 1st and 2nd cognitive interference tasks were analyzed after one participant was excluded due to a procedural error and replaced by an additional participant. For the correct ratio analysis, the mean percentage of correct responses was calculated in each condition (3 interventions: FAM vs. OMM vs. control × 2 timings: 1st vs. 2nd × 3 facial expressions: angry vs. smiling vs. neutral), and a 3 × 2 × 3 rm-ANOVA with one between-subject factor (intervention) and two within-subject factors (timing and facial expression) was conducted to examine the effect of interventions, timings, and facial expressions on the accuracy. For the reaction time analysis, trials with incorrect responses and reaction times longer than 1,000 ms were excluded. Furthermore, trials wherein the reaction time was longer or shorter than the mean ± 2 × intra-individual SDs for each participant for each condition were also excluded. On average, 5.70% of trials were excluded from the following analyses. Moreover, participants whose mean reaction time was longer or shorter than the mean ± 2 × inter-individual SDs in either of the cognitive interference tasks were excluded. Five participants were excluded and replaced by additional participants. The mean reaction time was analyzed by a 3 × 2 × 3 rm-ANOVA with the same factors as for the correct ratio.

In the preference judgment task, we assessed whether there was a mere exposure effect by calculating the difference between the preference of presented neutral face images and unpresented neutral face images in each condition. To perform this assessment, the mean preference was calculated for each condition (3 interventions: FAM vs. OMM vs. control × 2 exposure conditions: presented vs. unpresented in the 2nd cognitive interference task), and a 2 × 2 rm-ANOVA with one between-subject factor (intervention) and one within-subject factors (exposure condition) was conducted to examine the effect of interventions and exposure on preference.

In the surprise recognition task, recognition performance was assessed using A-prime (Aaronson and Watts, 1987) for each condition (intervention). A one-way ANOVA was conducted to examine the incidental learning during the 2nd cognitive interference task.

All statistical analyses were performed using SPSS 28.0.1.0 (IBM Corp., Armonk, NY, United States).

## 3 Results

### 3.1 Questionnaire

The results of the ANOVA of the trait questionnaires, including FFMQ, SPS, and LOT-R, for each group are shown in Table 1. There were no significant differences among the three intervention groups, *F*s < 0.91, *p*s > 0.41, *η*_p_^2^ < 0.003, indicating that there were no differences in the traits of mindfulness, social phobia, and optimism, all of which have the potential to influence the performance across all of the cognitive interference, preference judgment, and surprise recognition tasks. The results of the rm-ANOVA of state anxiety and relaxation between the 1st and 2nd state questionnaires for each group are shown in Tables 2, 3. The state anxiety level after intervention was lower than before, *F* (1, 69) = 30.18, *p* < 0.001, *η*_p_^2^ = 0.30, while the state relaxation level after the intervention was higher than before, *F* (1, 69) = 38.02, *p* < 0.001, *η*_p_^2^ = 0.36. This suggests that all interventions reduced anxiety levels and increased relaxation levels. Furthermore, there were no differences among groups in the state, both of which have the potential to influence the performance in the 2nd post-cognitive interference task.

**Table 1:** Results of the one-way ANOVA of trait questionnaires between groups.

	FAM		OMM		Control				
	*M*	SD	*M*	SD	*M*	SD	*F*	*p*	*η*_p_^2^
FFMQ									
Observing	2.69	0.68	2.70	0.54	2.65	0.58	0.05	0.95	0.00
Nonreactivity	2.64	0.56	2.79	0.56	2.81	0.62	0.60	0.55	0.02
Nonjudging	3.09	0.74	3.19	0.99	3.27	0.99	0.21	0.81	0.01
Describing	2.93	0.72	3.03	0.79	2.71	0.99	0.91	0.41	0.03
Act with awareness	3.29	0.77	3.18	0.68	3.04	0.66	0.79	0.46	0.02
Social Phobia Scale	0.78	0.50	0.97	0.60	0.85	0.45	0.81	0.45	0.02
LOT-R	2.99	0.44	3.10	0.57	3.00	0.49	0.40	0.67	0.01

FAM, focused attention meditation; OMM, open monitoring meditation; FFMQ, Five Facet Mindfulness Questionnaire; LOT-R, Life Orientation Test-Revised.

**Table 2:** Results of the rm-ANOVA of state anxiety between groups.

	FAM		OMM		Control		*F*	*p*	η_p_^2^
	*M*	SD	*M*	SD	*M*	SD			
STAI									
1st state questionnaire	1.84	0.35	1.88	0.37	1.80	0.26			
2nd state questionnaire	1.72	0.33	1.65	0.23	1.56	0.37			
Main effect of intervention							30.18	0.00	0.30
Main effect of group							0.87	0.43	0.03
Interaction							1.18	0.31	0.03

FAM, focused attention meditation; OMM, open monitoring meditation; STAI, State–Trait Anxiety Inventory.

**Table 3:** Results of the rm-ANOVA of state relaxation between groups.

	FAM		OMM		Control		*F*	*p*	η_p_^2^
	*M*	SD	*M*	SD	*M*	SD			
S-MARE									
1st state questionnaire	3.85	0.54	3.96	0.52	4.00	0.61			
2nd state questionnaire	4.23	0.43	4.29	0.47	4.48	0.45			
Main effect of intervention							38.02	0.00	0.36
Main effect of group							1.28	0.28	0.04
Interaction							0.40	0.67	0.01

FAM, focused attention meditation; OMM, open monitoring meditation; S-MARE, self-report measure to assess relaxation effects.

### 3.2 Cognitive interference task

The correct ratio and reaction time in the 1st and 2nd cognitive interference tasks are summarized in Table 4. For the reaction time, the rm-ANOVA showed significant main effects in timing, *F* (1, 69) = 8.31, *p* = 0.005, *η*_p_^2^ = 0.11, and facial expression, *F* (1, 69) = 3.50, *p* = 0.03, *η*_p_^2^ = 0.05, indicating that the reaction time was shorter after the intervention than before, and longer for the angry than for the neutral face images. The main effect of the intervention and all interactions was not significant, *F*s < 2.57, *p*s > 0.08, *η*_p_^2^ < 0.08. For the correct ratio, the rm-ANOVA did not show any significant main effects or interactions, *F*s < 2.23, *p*s > 0.11, *η*_p_^2^ < 0.04, indicating that there was no speed-accuracy trade-off.

**Table 4:** Correct ratio and reaction time for each condition.

		1st-cognitive interference task						2nd cognitive interference task					
		Angry		Neutral		Smiling		Angry		Neutral		Smiling	
		*M*	SD	*M*	SD	*M*	SD	*M*	SD	*M*	SD	*M*	SD
FAM	CR	0.99	0.02	0.99	0.02	0.99	0.02	0.99	0.01	0.99	0.01	0.99	0.01
	RT (ms)	471	28	470	24	474	33	453	31	452	32	456	31
OMM	CR	0.99	0.02	0.99	0.02	0.98	0.02	0.99	0.02	0.99	0.01	0.98	0.02
	RT (ms)	447	52	444	46	444	47	443	43	442	44	444	45
Control	CR	0.99	0.02	0.99	0.02	0.99	0.03	0.99	0.02	0.99	0.02	0.99	0.02
	RT (ms)	452	32	444	29	447	30	443	35	442	34	440	34

FAM, focused attention meditation; OMM, open monitoring meditation; CR, correct ratio; RT, reaction time.

### 3.3 Preference judgment task

**Figure 4:**
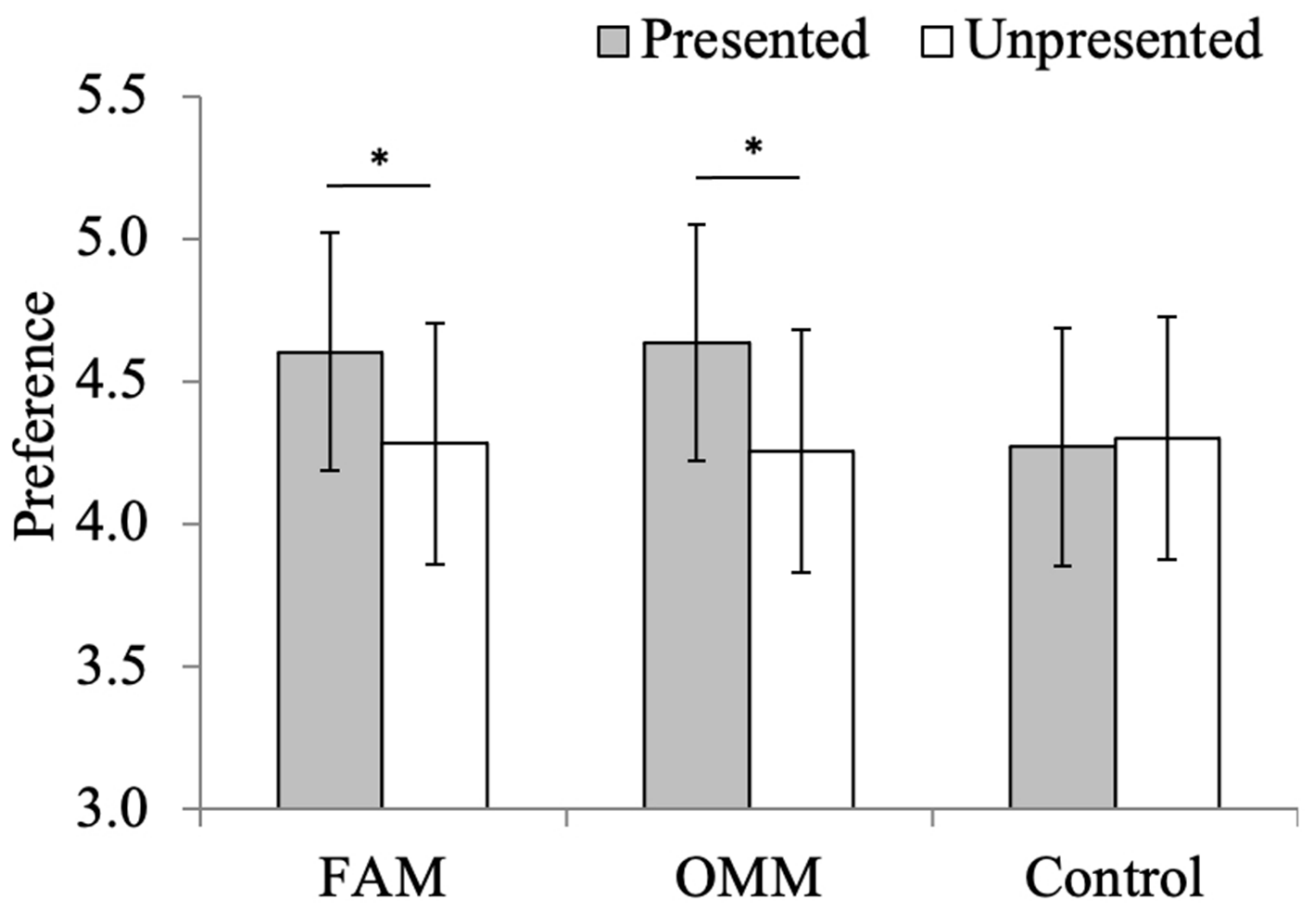
Average preference for each condition in the preference judgment task. FAM, focused attention meditation; OMM, open monitoring meditation. The error bar shows the 95% confidence interval, *p < 0.05.

The mean and standard deviation of preference for the presented and unpresented neutral face images in the preference judgment task in the FAM condition were 4.60 ± 1.04 and 4.28 ± 1.08, presented and unpresented neutral face images in the OMM conditions were 4.64 ± 0.94, 4.26 ± 1.02, and presented and unpresented neutral face images in the control conditions were 4.27 ± 1.06 and 4.30 ± 1.03, respectively (Figure 4). The rm-ANOVA revealed a significant main effect of exposure, *F* (1, 69) = 12.68, *p* < 0.001, *η*_p_^2^ = 0.16. The preference of the presented face images was higher than that of unpresented ones. Furthermore, an interaction between the intervention and exposure was significant, *F* (2, 69) = 4.19, *p* = 0.02, *η*_p_^2^ = 0.11. The simple main effects of exposure in the FAM and OMM groups were significant, *p* = 0.004 and *p* < 0.001, respectively, showing that the preference of the presented face images was higher than that of unpresented ones, indicating the mere exposure effect in the FAM and OMM groups. However, the simple main effect of exposure in the control group was not significant, *p* = 0.78, indicating that the mere exposure effect was not observed.

### 3.4 Surprise recognition task

In the surprise recognition task, the mean and standard deviation of A-primes of face recognition in the FAM, OMM, and control conditions were 0.56 ± 0.05, 0.60 ± 0.04, and 0.55 ± 0.04, respectively. The ANOVA did not show a significant main effect of the intervention, *F* (2, 69) = 0.36, *p* = 0.70, *η*_p_^2^ = 0.01.

### 3.5 Post-hoc analysis

Exploratorily, we investigated the relationship between the mere exposure effect and both state anxiety and relaxation after the intervention using Pearson correlation analysis. FAM entails the voluntary focusing of attention on a chosen object (Lutz et al., 2008), thereby inducing an increase in top-down attention regulation. The previous research indicated that stress could impair top-down attentional allocation and enhance stimulus-driven selection, leading to strong distractibility during a cognitive interference task (Sänger et al., 2014). Taking these into account, in the FAM group, it is expected that lower stress or higher relaxation states may facilitate concentration on the target and possibly reduce distractor inhibition in the cognitive interference task. It is also expected that higher stress or a lower relaxation state may impair concentration on the target and possibly increase distractor inhibition. Therefore, a negative correlation between state stress and the mere exposure effect or a positive correlation between state relaxation and the mere exposure effect may support the idea that FAM increases top-down attentional regulation. Meanwhile, OMM does not distinguish between selected and deselected objects (Lutz et al., 2008), therefore resulting in the absence of distractors to be inhibited. Therefore, it is expected that there exists no correlation between state stress or relaxation and the mere exposure effect.

**Figure 5:**
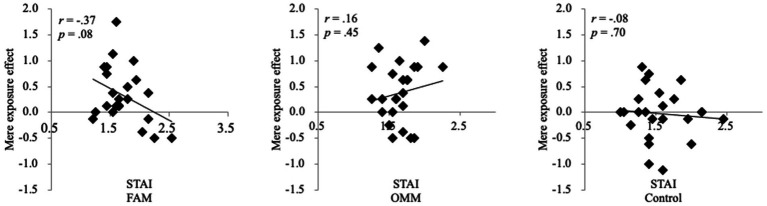
Correlation between the mere exposure effect and state anxiety. FAM, focused attention meditation; OMM, open monitoring meditation; STAI, State–Trait Anxiety Inventory.

**Figure 6:**
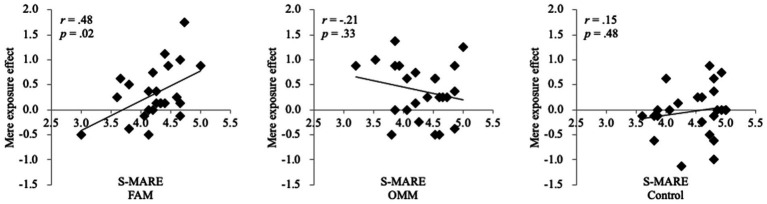
Correlation between the mere exposure effect and state relaxation. FAM, focused attention meditation; OMM, open monitoring meditation; S-MARE, self-report measure to assess relaxation effects.

As a result, there was a moderate negative correlation between the mere exposure effect and state anxiety in the FAM group, *r* = −0.370, *p* = 0.075, but not in the OMM and control groups, *r* = 0.16, *p* = 0.45, *r* = −0.08, *p* = 0.70, respectively (Figure 5). Furthermore, there was a positive correlation between the mere exposure effect and state relaxation in the FAM group, *r* = −0.48, *p* = 0.02, but not in the OMM and control groups, *r* = −0.21, *p* = 0.33, *r* = 0.15, *p* = 0.48, respectively (Figure 6). Consequently, the higher the state anxiety or the lower the state relaxation in the FAM group, the lower the mere exposure effect. No such relationship was observed in the OMM and control groups.

## 4 Discussion

In this study, we investigated the hypothesis that both FAM and OMM interventions would improve performance in the cognitive interference task compared to the control, and whether both FAM and OMM interventions would reduce the employment of a distractor inhibition strategy utilizing the mere exposure effect paradigm.

In the cognitive interference task, although reaction times became shorter after the intervention in every group, there were no significant group differences. Considering that the correct ratios for each condition consistently exceeded 0.98, the task was considered very easy, resulting in a ceiling effect.

To assess the reduction in the employment of a distractor inhibition, the mere exposure effect was observed in the FAM and OMM groups but not in the control group. In general, preference for objects increases after they have been repeatedly presented (Zajonc, 1968; Zajonc, 2001). Furthermore, previous research indicated that such preference was significantly higher in the case of exposure to various facial expressions compared with only a single facial expression (Kawakami and Yoshida, 2011). Indeed, in the preliminary experiment of this study, the preference for the face images increased significantly when the same face images were presented as targets, with the same presentation frequency and duration as in the current study (see more details about the preliminary experiment in the Supplementary material). However, in the control group of the current study, preference for the face images did not increase, despite sufficient exposure to face images with various expressions. Previous research indicated that when objects are presented as distractors in the cognitive interference task, such objects receive less positive or more negative preference due to distractor inhibition (De Vito et al., 2017; Fenske and Raymond, 2006; Huang and Hsieh, 2013; Inoue and Sato, 2017; Raymond et al., 2003; Raymond et al., 2005). Especially, Huang and Hsieh (2013) claimed that the absence of a negative rating for the non-attended stimuli does not necessarily mean that there was no distractor devaluation effect, because the negative distractor devaluation effect might have been overwhelmed by the positive mere exposure effect. Accordingly, the control group of the current study would inhibit face images during the cognitive interference task, resulting in a less positive preference for them. On the contrary, the mere exposure effect was observed in the FAM and OMM groups despite face images being distractors in the cognitive interference task. These results support the hypothesis that FAM and OMM reduce inhibition processes for distractors and use other attention regulation strategies.

Although identifying the underlying attention regulation strategy in FAM and OMM is beyond the scope of this study, post-hoc analysis may provide a cue, revealing a positive correlation between the mere exposure effect and state relaxation, as well as a negative correlation between the mere exposure effect and state anxiety after FAM, but not after OMM. This suggests that different attention regulation strategies may be employed between the two.

Previous research proposed that FAM enhances focusing attention on a target with top-down attention regulation (Lutz et al., 2008). The intervention instructions in the current study (Fujino et al., 2019), as well as traditional Buddhist meditation instruction (Hart, 1987), asked people to focus their attention on a specific object (i.e., target), but did not include instructions regarding distractor inhibition. Target facilitation and distractor inhibition depend on distinct cognitive mechanisms and neural substrates (Noonan et al., 2016; Xie et al., 2020), implying that both processes may occur simultaneously. Furthermore, computational cognitive modeling in the interference control indicated that FAM enhances controlled attention to goal-relevant targets rather than reducing automatic attentional activation to the goal-irrelevant distractor (Shields et al., 2020). Taken together, FAM may enhance target facilitation without distractor inhibition. However, the post-hoc analysis showing a positive correlation between the mere exposure effect and state relaxation, as well as a negative correlation between the mere exposure effect and state anxiety after FAM suggests that FAM may not always induce target facilitation without distractor inhibition. Based on these results, in the FAM group, participants with relatively higher levels of relaxation or lower levels of anxiety may enhance target facilitation without increasing distractor inhibition, resulting in a higher mere exposure effect. Conversely, participants with relatively lower levels of relaxation or higher levels of anxiety may enhance target facilitation with increasing distractor inhibition, resulting in a lower mere exposure effect. These results support the idea that FAM employs target facilitation.

It is proposed that OMM reduces the distinction between a target object and distractors (Lutz et al., 2008), suggesting that OMM may decrease distractor inhibition in comparison to the control. Our results demonstrating the mere exposure effect in the OMM group support this proposition. Furthermore, it is expected that OMM may decrease target facilitation in comparison to FAM, and our results showing the lack of correlation between the mere exposure effect and state relaxation, as well as state anxiety, support this proposition. In light of these findings, OMM may use different strategies other than enhancing distractor inhibition and target facilitation.

While this study successfully showed that FAM and OMM interventions in laypeople reduce inhibition processes for distractors at the behavioral level, there were some limitations. First, although both FAM and OMM interventions improved the reaction time in the cognitive interference task, considering that the task was very simple, this study found no difference in their influences on the reaction time of the cognitive interference task, resulting in a ceiling effect. In future studies, the effect of mindfulness meditation intervention on cognitive interference should be investigated with an increase in task difficulty. Second, the findings of this study were based on a 30-min meditation intervention on individuals with no prior meditation experience. Previous studies indicated that the influence of meditation on cognitive performance, physiological reactivity, and neural activity depends on the degree of meditation practice (Davidson and Dahl, 2018). In future studies, the influence of the degree of meditation practice on distractor inhibition should be investigated with individuals who have more extensive, long-term meditation experience.

In summary, the study found that FAM and OMM increased preferences even for distractors of a cognitive interference task, but relaxation did not. This indicates that FAM and OMM reduce inhibition processes for distractors during cognitive interference tasks and induce the mere exposure effect. Furthermore, there was a positive correlation between the intensity of the mere exposure effect and state relaxation, as well as a negative correlation between the former and state anxiety after FAM, but none after OMM. This suggests that FAM enhances target facilitation rather than distractor inhibition, and that OMM does not use inhibition but rather employs an attention regulation strategy differing from target facilitation and distractor inhibition. By refining the experimental design employed in this study, we elucidate a more comprehensive understanding of the neural, biological, and psychological mechanisms of mindfulness meditation and clarify the mechanism of the attention regulation strategy to improve performance in cognitive interference.

## Data Availability

The datasets presented in this study can be found in the following online repositories: https://osf.io/36rsq/?view_only=0d64d530b6f947ce94da1e7ce708c552.

## Supplementary material

SUPPLEMENTARY FIGURE 1 The image was reproduced from an existing publication in Ueda et al. (2019).
