# Supplementary material for: Prior brief meditation reduces distractor inhibition during cognitive interference
Source: Front Psychol. 2024 Oct 1;15:1445327. doi: 10.3389/fpsyg.2024.1445327 (PMC11473444; doi:10.3389/fpsyg.2024.1445327)
Supplement: SUPPLEMENTARY FIGURE 1 — The image was reproduced from an existing publication in Ueda et al. (2019). [file Data_Sheet_1.docx]

# *Supplementary Material*

# Preliminary Experiment: Examining the Influence of the Task on the Mere Exposure Effect

# Introduction

In this preliminary experiment, we examined whether the preference for face images increased when the same face images used in the main study were repeatedly presented as targets rather than distractors. The presentation frequency and duration were consistent with the main study. For this purpose, we prepared the cognitive interference task and non-cognitive interference task. For the cognitive interference task, a human face image was used as the distractor, and participants were asked to discriminate the orientation of a letter “T” (left or right) presented in between the eyes of the face image (Supplementary Figure 1A). For the non-cognitive interference task, a human face image was used as the target without any letters, and participants were asked to discriminate the sex of the face (female or male) (Supplementary Figure 1B).

We hypothesized that in the preference judgment task following the cognitive interference task, there would be no mere exposure effect when the face images are distractors, because participants tend to inhibit it. However, in the preference judgment task following the non-cognitive interference task, the mere exposure effect would be observed when the face images are not distractors but targets.

# Materials and Methods

# Participants

The study predetermined the sample size as 48, based on the necessity of counterbalancing and our laboratory’s experience. The required minimum sample size was 34 participants using G*Power (version 3.1.9.2; Faul et al., 2007) for an analysis of variance (ANOVA) (repeated measures, within-between interaction), using an effect size of 0.25, a significance level of 0.05, and a power of 0.8. To investigate the mere exposure effect with participants who appropriately completed the cognitive or non-cognitive interference tasks, we established exclusion criteria (mentioned in the data analysis section) and checked for them before analyzing the results of the preference judgment task. When a participant was excluded, we recruited another one. Fifty undergraduate and graduate students from Kyoto University, who were naïve to meditation practices, were recruited. We excluded one participant because that participant pressed the button inversely during the cognitive interference task and one participant due to reaction times (See Data analysis). Finally, data from 48 participants were analyzed (24 females, 24 males; *M_age_* = 23.10, SD = 4.07). Half were assigned to the cognitive interference task condition, and the other half to the non-cognitive interference task condition.

Before the experiment, written informed consent was obtained from all participants. All methods were performed according to the relevant guidelines and regulations of the institute and the Declaration of Helsinki. This study was approved by the internal ethics committee of the Graduate School of Education at Kyoto University (approval number: CPE-507).

# Apparatus

Instructions and visual stimuli were displayed on a monitor (Dell P992) with a resolution of 1,360 × 768 pixels using MATLAB (MathWorks, USA) using the Psychophysics Toolbox Version 3.0.12 (Brainard, 1997; Pelli, 1997). Participants sat on a chair with their heads positioned on a chin rest at a distance of approximately 40 cm from the monitor.

# Face images

Images of angry, neutral, and smiling faces of eight females and eight males were chosen from the Kokoro Research Center facial expression database (Ueda et al., 2019), and were classified into two sets of three facial expressions of four females and four males. All images in one set were used in cognitive or non-cognitive interference tasks as exposure stimuli. Furthermore, the neutral face images in both sets were used as the test stimuli in the following preference judgment task. Notably, while three types of facial expressions were used in the cognitive interference task, only neutral faces were used in the preference judgment task. This design was based on previous research indicating that preference was significantly higher when participants were exposed to various facial expressions compared to a single facial expression (Kawakami and Yoshida, 2011). The same neutral face images were also used as test stimuli in the subsequent surprise recognition task, which aimed to confirm whether participants recognized the face images during the cognitive interference task. The size of all face images was 19.2° in width and 22.6° in height. The face image sets were counterbalanced across participants.

# Procedure

Participants were randomly assigned to two groups—the cognitive interference task group or non- cognitive interference task group, each of which consisted of 24 participants. Experimenters that were unaware of the study’s purpose did not know which condition the participants were assigned to because the experimenters only pressed the button to start the prepared program for the experiment. Participants did not know what kind of conditions they were involved in either (i.e., double-blind). Participants performed either the cognitive or non-cognitive interference task, followed by the preference judgment and surprise recognition tasks.

In the cognitive interference task, a face image was used as a distractor, while a letter (“T”) placed between the eyes of the face was used as a target (supplementary Figure 1A). In the beginning of the trial, the fixation appeared at the center of the display for 1000, 2000, or 3000 ms randomly; subsequently, the letter and face image appeared. Participants were asked to discriminate the orientation of the letter (left or right) and answer it by using the keyboard as soon and accurately as possible while ignoring the facial expressions. Both the letter and face image were presented for 660 ms irrespective of the participants’ key press. In the non-cognitive interference task, a face image was used as a target, while the letter (“T”) was not presented (Supplementary Figure 1B). Other stimulus presentation schedules were the same as the cognitive interference task. Participants were asked to discriminate the sex of the face images (female or male) and answer it using a keyboard as soon and accurately as possible. For both tasks, there were six practice trials followed by seven experimental blocks, each comprising 24 trials. In each block, the three facial expressions of four females and four males were presented once in random order. Therefore, participants were exposed to each person’s face 21 times (i.e., three facial expressions × seven blocks).

In the preference judgment and surprise recognition tasks, after the fixation appeared in the center of the display for 1000 ms, a neutral face appeared. In the preference judgment task, participants were asked to rate their preference on a nine-point Likert scale (1 = very unattractive, 5 = neutral, and 9 = very attractive). In the following surprise recognition task, they judged whether it was presented in the cognitive or non-cognitive interference task. The face image was presented until the participant responded. There were 16 trials conducted; half were presented in the cognitive or non-cognitive interference task and the other half were novel to the participants. Participants were given no information about the preference judgment task until they had finished the cognitive or non-cognitive interference task and about the surprise recognition task until they finished the preference judgment task.

# Data Analysis

In the cognitive and non-cognitive interference tasks, the correct ratio and reaction time were analyzed. For the correct ratio analysis, the mean percentage of correct responses was calculated in each condition (2 tasks: cognitive interference task vs. non-cognitive interference task × 3 facial expressions: angry vs. smiling vs. neutral), and a 2 × 3 repeated-measures analysis of variance (rm-ANOVA) with one between-subject factor (task) and one within-subject factor (facial expression) was conducted to examine the effect of tasks and facial expressions on the accuracy. For the reaction time analysis, trials with incorrect responses and reaction times longer than 1000 ms were excluded. Furthermore, trials wherein the reaction time was longer or shorter than the mean ± 2 × intra-individual SDs for each participant for each condition were also excluded. On average, 7.07% of the cognitive interference task trials and 30.90% of non-cognitive interference task trials were excluded from the following analyses. Moreover, participants whose mean reaction time was longer or shorter than the mean ± 2 × inter-individual SDs for each cognitive task and non-cognitive interference task were excluded. Two participants were excluded and replaced by testing additional participants. The mean reaction time was analyzed by a 2 × 3 rm-ANOVA with the same factors as for the correct ratio.

In the preference judgment task, we assessed whether there was a mere exposure effect by calculating the difference between the preference of presented neutral face images and unpresented neutral face images in each condition. To perform this assessment, the mean preference was calculated for each condition (2 tasks: cognitive interference task vs. non-cognitive interference task × 2 exposure conditions: presented vs. unpresented in the cognitive or non-cognitive interference tasks), and a 2 × 2 rm-ANOVA with one between-subject factor (task) and one within-subject factor (exposure condition) was conducted to examine the effect of tasks and exposure on preference.

In the surprise recognition task, recognition performance was assessed using A-prime (Aaronson and Watts, 1987) for each condition (2 tasks). An unpaired t-test of A-primes between the cognitive and non-cognitive interference tasks was conducted to examine the incidental learning during their tasks.

All statistical analyses were performed using SPSS 28.0.1.0 (IBM Corp., Armonk, NY, United States).

# Results

# Discrimination Task

The correct ratio and reaction time in the cognitive interference and non-cognitive interference tasks are summarized in Supplementary Table 1.

For reaction time, the rm-ANOVA showed a significant main effect, *F* (1, 46) = 126.67, *p* < .001, η_p_^2^ = .73, indicating that it was shorter in the cognitive interference task than in the non-cognitive interference task. The main effect of facial expression was not significant, *F* (2, 92) = 0.69, *p* = .50, η_p_^2^ = .02, and neither was the interaction, *F* (2, 92) = 0.65, *p* = .53, η_p_^2^ = .01.

For correct ratio, the rm-ANOVA showed significant main effects of the tasks, *F* (1, 46) = 135.98, *p* < .001, η_p_^2^ =.75, and facial expressions, *F* (2, 92) = 25.67, *p* < .001, η_p_^2^ = .36. The correct ratio of the cognitive interference task was higher than that of the non-cognitive interference task, indicating that there was no speed-accuracy trade-off. Moreover, the correct ratio of trials with the angry face image was lower than that of neutral and smiling face images. Furthermore, there was a significant interaction between the task and facial expression, *F* (2, 92) = 20.56, *p* < .001, η_p_^2^ =.31. The simple main effect of facial expression was not significant in the cognitive interference task, *F* (2, 92) = 0.29, *p* = .75, η_p_^2^ = .01, but was significant in the non-cognitive interference task, *F* (2, 92) = 33.08, *p* < .001, η_p_^2^ = .60. Multiple comparisons with Bonferroni correction revealed that the accuracy of trials with the angry face image was lower than that of the neutral and smiling face images, *p*s < .001.

# Preference Judgment Task

The mean and standard deviation of preference for the presented and unpresented neutral face images in the cognitive interference task were 3.92 ± 0.85 and 3.98 ± 0.82, respectively, while they were 4.67 ± 0.67 and 4.38 ± 0.72 in the non-cognitive interference task, respectively (Supplementary Figure 2). The rm-ANOVA revealed a significant main effect of the tasks, *F* (1, 46) = 7.87, *p* =.001, η_p_^2^ = .15, indicating that preference after the non-cognitive interference task was rated higher than that after the cognitive interference task. The main effect of the exposure condition was not significant, *F* (1, 46) = 1.88, *p* = .18, η_p_^2^ = .04. Furthermore, their interaction was significant, *F* (1, 46) = 4.48, *p* = .04, η_p_^2^ = .09. The simple main effect of the exposure condition under the non-cognitive interference task was significant, *F* (1,46) = 6.08, *p* = .02, η_p_^2^ = .12, showing that the preference of presented face images was rated higher; that is, the mere exposure effect was observed. The simple main effect of the exposure condition under the cognitive interference task was not significant, *F* (1,46) = 0.28, *p* = .60, η_p_^2^ = .01, indicating that the mere exposure effect was not observed.

# Surprise Recognition Task

In the surprise recognition task, the mean and standard deviation of the A-prime of face recognition in the cognitive interference task condition were 0.58 ± 0.19, and those in the non-cognitive interference task condition were 0.84 ± 0.13. The unpaired *t*-test showed a significant difference between tasks, *t* (46) = 5.57, *p* < .001, Cohen’s *d* = 1.61. The recognition performance of the face images in the cognitive interference task was lower than in the non-cognitive interference task.

# Discussion

The main purpose of this preliminary experiment was to examine whether the preference for the face images increased when they were repeatedly presented as a target or distractor, with the same presentation frequency and duration. We found the mere exposure effect when they were presented as targets (i.e., the non-cognitive interference task). In contrast, no such effect was observed when they were presented as distractors (i.e., the cognitive interference task). The main difference between the cognitive and non-cognitive interference tasks was whether face images were the target or distractor that participants should focus on to perform tasks. Previous research indicated that when objects are presented as distractors in the cognitive interference task, they receive less positive or more negative preference due to distractor inhibition (De Vito et al., 2017; Fenske and Raymond, 2006; Huang and Hsieh, 2013; Inoue and Sato, 2017; Raymond et al., 2003; Raymond, Fenske, and Westoby, 2005). Taken together, it could be interpreted that the positive preference observed in the non-cognitive interference task disappeared in the cognitive interference task due to distractor inhibition.

# References

Aaronson, D., and Watts, B. (1987). Extensions of Grier’s computational formulas for A’ and B” to below-chance performance. Psychol. Bull. 102(3), 439–442. [doi: 10.1037/0033-2909.102.3.439](https://doi.org/10.1037/0033-2909.102.3.439)

Brainard, D. H. (1997). The psychophysics toolbox. Spat Vis. 10(4), 433–436. [doi: 10.1163/156856897X00357](https://doi.org/10.1163/156856897X00357)

De Vito, D., Al-Aidroos, N., and Fenske, M. J. (2017). Neural evidence that inhibition is linked to the affective devaluation of distractors that match the contents of working memory. Neuropsychologia. 99, 259–269. [doi: 10.1016/j.neuropsychologia.2017.03.022](https://doi.org/10.1016/j.neuropsychologia.2017.03.022)

Faul, F., Edgar, E., Lang, A.-G., and Buchner, A. (2007). G*Power 3: A flexible statistical power analysis program for the social, behavioral, and biomedical sciences. Behav. Res. Methods. 39(2), 175–191. [doi: 10.3758/BF03193146](https://doi.org/10.3758/BF03193146)

Fenske, M.J., Raymond, J.E., 2006. Affective influences of selective attention. Curr. Dir. Psychol. Sci. 15(6), 312–316. [doi: 10.1111/j.1467-8721.2006.00459.x](https://journals.sagepub.com/doi/10.1111/j.1467-8721.2006.00459.x)

Huang, Y.-F., and Hsieh, P.-J. (2013). ﻿The mere exposure effect is modulated by selective attention but not visual awareness. Vis. Res. 91, 56–61. [doi: 10.1016/j.visres.2013.07.017](https://doi.org/10.1016/j.visres.2013.07.017)

Inoue, K., and Sato, N. (2017). ﻿Valuation of go stimuli or devaluation of no-go stimuli? Evidence of an increased preference for attended go stimuli following a go/no-go task. Front. Psychol. 8, 474. [doi: 10.3389/fpsyg.2017.00474](https://doi.org/10.3389/fpsyg.2017.00474)

Kawakami, N., and Yoshida, F. (2011). Multiple mere exposure effect: Category evaluation measured in the Go/No-go Association Task (GNAT). Jpn. J. Psychol. 82(5), 424-432. [doi: 10.4992/jjpsy.82.424](https://www.jstage.jst.go.jp/article/jjpsy/82/5/82_5_424/_article/-char/ja/)

Pelli, D. G. (1997). The VideoToolbox software for visual psychophysics: transforming numbers into movies, Spat. Vis. 10(4), 437–442. [doi: 10.1163/156856897X00366](https://doi.org/10.1163/156856897X00366)

Raymond, J. E., Fenske, M. J., and Tavassoli, N. T. (2003). Selective attention determines emotional responses to novel visual stimuli. Psychol. Sci. 14(6), 537–542. [doi: 10.1046/j.0956-7976.2003.psci_1462.x](https://doi.org/10.1046/j.0956-7976.2003.psci_1462.x)

﻿Raymond, J. E., Fenske, M. J., and Westoby, N. (2005). Emotional devaluation of distracting patterns and faces: A consequence of attentional inhibition during visual search? J. Exp. Psychol. Hum. Percept. Perform. 31(6), 1404–1415. [doi: 10.1037/0096-1523.31.6.1404](https://psycnet.apa.org/doiLanding?doi=10.1037%2F0096-1523.31.6.1404)

Ueda, Y., Nunoi, M., and Yoshikawa, S. (2019). Development and validation of the Kokoro Research Center (KRC) facial expression database, Psychologia. 61(4), 221–240. [doi: 10.2117/psysoc.2019-A009](https://doi.org/10.2117/psysoc.2019-A009)

# Figure Legends

**Supplementary Figure 1.** The Flow of the Cognitive Interference Task (A) and the non-Cognitive Interference Task (B)

A B

Note: When character “T” is a target, the face image would be a distractor (A). Conversely, the face image is a target when judging the sex of the face image (B). The image was reproduced from an existing publication in Ueda et al. (2019).

**Supplementary Figure 2.** Average Preference for Each Condition in the Preference Judgment Task

Note: The error bar shows the 95 % confidence interval. * *p* < 0.05

# Tables

Supplementary Table 1. Correct Ratio and Reaction Time for Each Condition

|  | Cognitive interference task | | | | | | | |  | Non-cognitive interference task | | | | | | | |
| --- | --- | --- | --- | --- | --- | --- | --- | --- | --- | --- | --- | --- | --- | --- | --- | --- | --- |
|  | Angry | |  | Neutral | |  | Smiling | |  | Angry | |  | Neutral | |  | Smiling | |
|  | M | SD |  | M | SD |  | M | SD |  | M | SD |  | M | SD |  | M | SD |
| Correct ratio | .97 | .03 |  | .98 | .03 |  | .97 | .03 |  | .67 | .13 |  | .81 | .09 |  | .82 | .09 |
| Reaction time (ms) | 418 | 33 |  | 416 | 33 |  | 418 | 32 |  | 580 | 75 |  | 584 | 61 |  | 587 | 65 |
